# Plasticity in metabolism underpins local responses to nitrogen in *Arabidopsis thaliana* populations

**DOI:** 10.1002/pld3.186

**Published:** 2019-11-29

**Authors:** Prashant K. Pandey, Jing Yu, Nooshin Omranian, Saleh Alseekh, Neha Vaid, Alisdair R. Fernie, Zoran Nikoloski, Roosa A. E. Laitinen

**Affiliations:** ^1^ Max Planck Institute of Molecular Plant Physiology Potsdam Germany; ^2^ Bioinformatics Institute of Biochemistry and Biology University of Potsdam Potsdam Germany

**Keywords:** *Arabidopsis thaliana*, natural variation, nitrogen availability, photorespiration, plasticity

## Abstract

Nitrogen (N) is central for plant growth, and metabolic plasticity can provide a strategy to respond to changing N availability. We showed that two local *A. thaliana* populations exhibited differential plasticity in the compounds of photorespiratory and starch degradation pathways in response to three N conditions. Association of metabolite levels with growth‐related and fitness traits indicated that controlled plasticity in these pathways could contribute to local adaptation and play a role in plant evolution.

## 1 INTRODUCTION

Nitrogen (N) is required for the synthesis of nucleotides, amino acids, and proteins, and is therefore central for plant growth and reproduction. Roots uptake N from soil, mainly in forms of nitrate and ammonium, which is then transported from root to shoot where it is metabolized (Hachiya & Sakakibara, 2017; Miller & Cramer, 2005; Nunes‐Nesi, Fernie, & Stitt, 2010). In natural environments, N availability is often limiting plant growth (Kiba & Krapp, 2016; Verkroost & Wassen, 2005). Therefore, it may be expected that plants have evolved different strategies to cope with changes in N availability. Both carbon and photorespiratory metabolism are known to interact with N assimilation and metabolism (Nunes‐Nesi et al., 2010; Rachmilevitch, Cousins, & Bloom, 2004; Stitt & Krapp, 1999) but it is not yet known how and to what extent these interactions are modulated in plant populations in nature.

N availability can be highly variable across different environments, prompting the question of how the corresponding responses are mediated (Lark et al., 2004). Plasticity, an ability of an organism to change its phenotype in different environments, could help plants to optimize their growth under changing N conditions. Plasticity in root system architecture has been linked to different N availabilities (Drew, Saker, & Ashley, 1973; Forde, 2014; Giehl, Gruber, & Wiren, 2014; Gifford, Dean, Gutierrez, Coruzzi, & Birnbaum, 2008), and recently natural variation in *A. thaliana* was used to study genetic cause of changes in root architecture in response to N (De Pessemier, Chardon,Juraniec, Delaplace, & Hermans, 2013; Jia, Giehl, Meyer, Altmann, & Wiren, 2019). However, not as much is known about metabolic plasticity and its role in plant growth in local *A. thaliana* populations adapted to different habitats.

We hypothesized that differential plasticity in pathways at the interface between carbon and N metabolism underpins the adjustment of plant populations toward optimal fitness at different N availabilities. To test the hypothesis, we analyzed plasticity to N availability in 65 primary metabolites as well as growth and fitness traits in two *A. thaliana* populations, one collected from Southern Germany and the other from Northern Sweden. These populations originate from different growth habitats and differ genetically (Plotner et al., 2017; Swiadek et al., 2017). Interestingly, we found that glutamine, an amino acid central to the assimilation of N, exhibited the highest plasticity in both populations. Furthermore, glycine, serine, glycerate, and maltose showed significantly different plasticity between the two populations. These findings indicated that the response to changes in N availability is facilitated by high population‐independent plasticity of glutamine and population‐dependent plasticity associated with photorespiratory metabolism and starch degradation.

## 2 MATERIALS AND METHODS

### 2.1 Plant conditions and phenotyping

Fifteen individual plants collected from Lövvik (Lov), Northern Sweden in year 2015 (Plotner et al., 2017), and 15 individual plants from Altenriet (Alt), Southern Germany in 2013 (Swiadek et al., 2017), were used in this study. In all conditions, soil contained 50% (v/v) fine white peat (Gramoflor GmbH) mixed with 30% (v/v) fine and 25% (v/v) coarse‐grained vermiculite peat (Fitz Kausek GmbH & Co). In the limited N condition, 2.6 g K_2_HPO_4_, 3.96 g GRANUKAL 85 (80% CaCO_3_ & 5% MgCO_3_—Dammann GmbH & Co), and 10.6 mg Fetrilon‐Combi micronutrient fertilizer (BASF AG) were mixed with 1 L of soil. In the intermediate N condition, 5.4 mg solid NH_4_NO_3_, and in the optimal conditions, 54.4 mg solid NH_4_NO_3_ were added per liter of soil mixture. To homogenize, the soil mixture was placed at 10℃ and mixed every second day for 2 weeks. Before sowing the seeds, each pot was weighed to contain equal amount of soil. After this, 8 replicates in individual pots for each condition were vernalized for 10 weeks at 4℃ in their specific soil and grown in growth chambers (Clf Plantclimatics GmbH) at 21℃/17℃ (light/dark) in long day (LD) (16 hr/8 hr) with 150 µM cm^−2^ s^−1^ conditions. Vernalization equalizes the flowering time, and all accessions in both populations started to flower between 15 and 19 days after moving them to the growth chambers. Four replicates were photographed every third day, and ImageJ (https://imagej.nih.gov/ij/) was used to calculate the rosette diameter (RD) from the photographs. The final rosette diameter (FRD) was measured at the time of bolting. Growth rate (GR) was calculated as (Final RD − RD day 1)/days. For total seed yield, plants were let to senesce naturally, and when no open flowers were observed, the plants were covered with paper bags. Seeds of two fully dried plants were collected into one tube. Then, 100 seeds from two replicates (2 × 100), each pooled from two plants, were counted and weighed. To calculate the total seed number, the total seed weight was divided by mean weight of the 2 × 100 seeds and multiplied by 100.

### 2.2 Genetic and metabolic analysis

For the genetic analysis, the RADseq data (Plotner et al., 2017 and Swiadek et al., 2017) were used to investigate the genetic differences among populations. First, RADseq data were filtered using 10× coverage in all samples and to contain at least one polymorphic SNP among the analyzed accessions. The resulting 2,171 SNPs among the populations were assigned as informative and were used for further analysis. For percentage (%) of sequence similarity, these SNPs were compared to each other using MEGALIGN. Degree of heterozygosity (He index) was calculated using “pegas” in R package. For metabolic analysis, next generation of seeds from the genotyped accessions were used. To avoid any bias due to different flowering times, whole rosettes of plants were harvested before flowering at 10‐leaf stage in four replicates per accession and used in the metabolic analysis. The sampling was carried out in the mid‐day (between 12:00–14:00) to avoid bias caused by circadian effects. Extraction and analysis of 65 primary metabolites were performed according to Lisec et al. (2011). The GC‐MS system used was a gas chromatograph coupled to a time‐of‐flight mass spectrometer (Leco Pegasus HT TOF‐MS). An auto sampler Gerstel MultiPurpose system injected the samples. Chromatograms and mass spectra were evaluated by using Chroma TOF 4.5 (Leco) and TagFinder 4.2 software (Roessner et al., 2001; Schauer et al., 2005). Metabolites were evaluated on the basis of the peak area ion peaks processed using Xcalibur 2.1 software (Thermo Fisher Scientific). The obtained relative peak intensity was normalized by comparison to an internal standard (ribitol; CAS488‐81‐3) and the fresh weight of the sample used for extraction.

### 2.3 Data analysis

**Figure 1:**
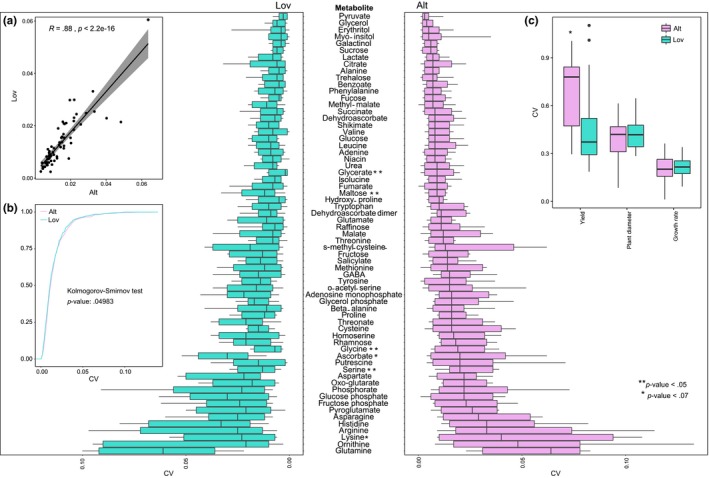
Plasticity in the 65 primary metabolites, two growth and one fitness trait in *A. thaliana* populations Lövvik (Lov) and Altenriet (Alt). Metabolites are ordered according their median coefficient of variation (CV) values in the Alt population. The CVs of metabolite levels are significantly correlated between the two populations (inlay a, *R* = 0.88), with the Alt population exhibiting larger CVs in comparison to the Lov population (inlay b, Kolmogorov–Smirnov (KS) test). There are significant differences in the CVs of the rosette diameter and growth rate as well as yield between the two populations (inlay c). Metabolites showing significantly different plasticity between populations (one‐way ANOVA, *p*‐value .05 adjusted by the Benjamini–Hochberg procedure) are indicated with asterisk(s)

The means for each trait over the four replicates (65 for metabolites, four replicates for rosette diameter and yield) for each accession were first determined for each N condition (Figure S1). The CV for a trait of an accession was then determined by calculating the mean and standard deviation for the three means of the N conditions and then taking the ratio standard deviation and the mean. The plasticity of the trait in the population can then be characterized by the mean of the CVs over the 15 accessions (Figure 1, main text). The differences between plasticity of populations were tested by one‐way ANOVA for each trait separately. The difference between the distributions of CVs and metabolite level distributions over all traits were tested by using the Kolmogorov–Smirnov test implemented in R. Pearson correlation coefficients for all analyzed traits were determined for each of the population separately using the built‐in function in R. All reported *p*‐values are corrected with the Benjamini–Hochberg procedure implemented in a standard R function.

## 3 RESULTS AND DISCUSSION

In addition to the global *A. thaliana* accessions, few reports have also investigated plasticity in phenotypic traits in wild *A. thaliana* populations (Callahan & Pigliucci, 2002; Pigliucci & Schlichting, 1996; Pigliucci, Schlichting, & Whitton, 1995; Pigliucci, Whitton, & Schlichting, 1995). Yet, how phenotypic plasticity in wild *A. thaliana* populations reflects to their genetic diversity is not known. We first asked if the degree of plasticity in the *A. thaliana* populations was associated with the degree of genetic diversity. To answer this question, we grew 15 *A.* *thaliana* accessions from two populations originating from different growth habitats on three N conditions tested to be limiting, intermediate, and optimal for growth. While the accessions in Northern Sweden in Lövvik (Lov) were collected from an undisturbed south‐facing slope, the accessions in Southern Germany in Altenriet (Alt) were collected from a field side with frequent human disturbance. We analyzed plasticity in 68 traits, including the levels of 65 compounds from primary metabolism (i.e., 25 amines, 19 acids, 12 sugars, and 4 phosphates) as well as rosette diameter and growth rate, as two growth‐related traits, and seed yield, as a fitness trait (Figure S1). Genetic analysis showed that Lov accessions are genetically very similar (>98.7%) with only five polymorphic SNPs out of 2,171 informative SNPs ((Plotner et al., 2017), Table S1), while Alt accessions are more different (i.e., genetic similarity between 65% to 91%) ((Swiadek et al., 2017), Table S1). Four out of the five polymorphic SNPs in Lov population were heterozygous leaving only a single polymorphic nucleotide in only one of the individual. The overall heterozygosity in Alt population was low (He = 0.199), but higher than in Lov population (He = 0.009). We note that seeds used in this study are the next generation from the selfed individuals genotyped, and in these, the heterozygous loci are segregating yet influencing the genetic diversity. Nevertheless, the Alt population was genetically more diverse than in the Lov population. To quantify plasticity for each trait of an accession, we first calculated the mean trait value for the four replicates separately for the three N conditions. Then, we determined the coefficient of variation (CV) of the mean values for each of the 68 traits over the three N conditions and used it as a measure of plasticity of the trait for each accession. By comparing the distributions of CVs over all traits between the two populations, we found that the accessions in the genetically more diverse Alt population showed higher range of plasticity and were on average also more plastic than the individuals in the Lov population (Figure 1 a, b, *p*‐value < .05, KS‐test). Together with the lack of plasticity in majority of metabolites, also the reaction norms of majority of the traits were similar between the two populations (Figure S1).

Next, we asked if the two populations differed in the metabolic strategies used to cope with the different N availability. To this end, we compared the average amount of metabolic plasticity among the individuals between the two populations (Figure 1). We note that our findings are based on metabolomics data obtained from plant material harvested in the middle of the day. Therefore, our analysis of plasticity does not take into account possible differences in the operation of the clock between the accessions. The latter can be addressed in future studies by conducting time‐resolved sampling, although it will require different type of data aggregation. From the CVs of the 65 metabolites, expectedly 61 did not show significant differences between populations since most of the primary metabolites were highly robust across the three nitrogen conditions (CV < 5%). Interestingly, while in most cases the similar CV is a result of similar response; in 18 metabolites, the distributions of the metabolite levels were significantly different between the populations (KS‐test, adjusted *p*‐value < .05; Table S2). Furthermore, when we compared the CVs of the different classes of metabolites (amines, acids, phosphates, and sugars) and we found that the nitrogen containing metabolic classes, phosphate, and amines showed the highest plasticity while sugars were the least variable (Figure S2). Interestingly, the levels of glutamine exhibited the highest plasticity in both communities, with an average CV of 6.4% and 6.1% in the Alt‐ and the Lov‐community, respectively (Figure 1). Glutamine levels are reported to differ among genotypes and environments and show plasticity in response to diurnal cycle (Forde & Lea, 2007; Stitt & Fernie, 2003). Our result indicated that, despite the genetic differences between the two populations, and the limited genetic variation in the Lov population, both populations adjust their glutamine levels in response to differences in N availability. Further, we found that the three metabolites participating in photorespiration, glycine, serine, and glycerate, exhibited on average significantly higher plasticity in Alt‐ in comparison to Lov population (Figure 1). Plants can acquire N from soil by fixing ammonium into glutamine by glutamine synthetase (Hachiya & Sakakibara, 2017). Photorespiration, in turn, releases ammonium which can be re‐assimilated using glutamine synthase that provides a link between photorespiration and N metabolism. Such a link between carbon and N metabolism could be beneficial for plants by increasing the CO_2_ uptake and diminishing some of the negative effects of photorespiration on plant growth (Busch, Sage, & Farquhar, 2018). In contrast, maltose, a major product of starch degradation at night, showed significantly lower plasticity in the Alt‐ in comparison to the Lov population. Remobilization of starch during the night is used as a carbon source for growth and plays a role in determining plant fitness (Lu & Sharkey, 2006; Thalmann & Santelia, 2017). These results suggest that accessions in the Alt population adjust photorespiratory metabolism to maintain growth, while those in the Lov population modulate starch degradation in response to different N availabilities. It remains to be studied in future whether these changes are linked to specific genetic differences between populations.

If the plasticity of metabolite levels contributed to local adaptation, we would expect to see that the mean metabolite levels correlate significantly with the growth and fitness traits in each population. Interestingly, we found significant negative correlation between glutamine levels and rosette diameter (adjusted *p*‐value = .0002) and growth rate (adjusted *p*‐value = .0015) in the Lov population (Table 1). The correlations were also negative in the Alt population, but not significant after multiple hypotheses testing correction. This observation was in line with the expectation that the majority of the acquired N is mobilized toward growth, rather than transiently stored in the glutamine pool. In addition, both serine and glycine showed significant positive correlation with rosette diameter, while glycine also exhibited significant positive correlation with the growth rate in both populations (adjusted *p‐*values < .05) (Table 1). Further, glycine was the only metabolite with differential plasticity which showed significant positive correlation with yield, only in the Alt population. These findings provided further evidence that different strategies based on modulating the link between photorespiration and N metabolism are used by the two populations.

**Table 1:** Correlation analysis between metabolite levels, growth, and fitness traits

		Alt		Lov	
		correlation	*p*‐value	correlation	*p*‐value
Metabolites showing significant correlations to complex traits in both populations					
Fumarate	Plant diameter	.560495519	0.000706984	.507937872	.001376011
Fumarate	Growth rate	.433545006	.014299358	.400917167	.015093554
Glycerol phosphate	Growth rate	−.378500146	.037021187	−.447500397	.005795752
Glycine	Growth rate	.370013042	.042414418	.368253207	.027588709
Pyruvate	Growth rate	.365012479	.045835444	.341318183	.043321798
Pyruvate	Yield	.363666344	.046700914	.507858621	.001376339
Homoserine	Plant diameter	−.362716831	.047246927	−.336390862	.046693947
Glycine	Plant diameter	.423000321	.017226176	.466089339	.003853872
Metabolites showing significant correlations to complex traits only in the Alt population					
Fumarate	Yield	.53997473	.001262268	.117471438	.537847395
Nicotinate	Plant diameter	.496540744	.003858711	.237010869	.179986583
Tryptophan	Yield	.469055581	.007041944	.055809918	.783205713
Dehydroascorbate	Plant diameter	.443629257	.011537902	.196875846	.275484303
*Myo‐insitol*	Plant diameter	.427120937	.016152696	.035546973	.865804471
Dehydroascorbate dimer	Plant diameter	.410073872	.021828548	.1093866	.570648134
*Myo‐insitol*	Yield	.405282533	.023806197	.089084056	.647960157
Glycine	Yield	.392184985	.029535749	.146849127	.428960484
Dehydroascorbate	Yield	.390862155	.030021473	.245974438	.162367779
Phenylalanine	Yield	.371840462	.041014881	−.000299495	.998442104
Dehydroascorbate	Growth rate	.363292716	.046888445	.13632786	.46814536
Metabolites showing significant correlations to complex traits only in the Lov population					
Glycerol phosphate	Plant diameter	−.33622214	.069647921	−.4693648	.003561781
Glutamine	Plant diameter	−.318948071	.08743339	−.569863933	.000232486
Glucose	Plant diameter	.313044404	.094410106	.410513424	.012598708
Oxoglutarate	Yield	.304894673	.104756582	.384637876	.020598852
Glucose phosphate	Plant diameter	−.278939037	.147120775	−.350463457	.037602199
Histidine	Growth rate	−.234917828	.236768322	−.375080821	.02439994
Histidine	Plant diameter	−.216210663	.28233485	−.364488036	.029504793
Proline	Growth rate	.193498653	.34856502	−.410751686	.012554147
Glucose	Growth rate	.181802462	.384078946	.406308612	.013598452
Glutamine	Growth rate	−.177031412	.397737598	−.504131785	.001518398
Lysine	Plant diameter	−.151848199	.477963364	−.46571305	.003874617
Leucine	Growth rate	.087979132	.70228349	−.44329505	.006342394
Leucine	Plant diameter	.080125783	.734404971	−.444975164	.006130827
Lysine	Growth rate	−.075796806	.747300298	−.440374152	.006778191
Gaba	Plant diameter	.075016411	.750194656	−.340402231	.043900206
Aspartate	Plant diameter	−.074938711	.750264524	−.340074832	.044136168
Adenosine monophosphate	Growth rate	−.072763602	.759841738	−.408228865	.013151936
Isoleucine	Plant diameter	.046172975	.855578058	−.332033	.049970872
Aspartate	Growth rate	−.046144619	.855578058	−.350920005	.037425414
Adenosine monophosphate	Plant diameter	−.044323128	.86238426	−.447102713	.005842538
Isoleucine	Growth rate	.042893974	.864925917	−.36770522	.027875485
Gaba	Growth rate	.035978367	.886947295	−.401661288	.014870376

Note

Pearson correlation coefficients determined for both populations between metabolites and yield, plant diameter, and growth rate and p‐values adjusted for multiple hypotheses testing

Maintenance of plasticity of fluxes (and their efficiencies) is the key to increase growth. Under the assumption that enzymes operate far from substrate saturation, and their levels do not change as fast as those of metabolites, plasticity of metabolites can be used as a proxy for the plasticity of fluxes. Our study showed that although plant primary metabolism is highly robust to changes in N availability, maintenance of plasticity in certain metabolic pathways is key to increase growth and fitness in local populations. Indeed, the correlation of the mean levels of the metabolites with high plasticity and growth traits indicated that metabolic plasticity could provide an advantage for plant populations to cope with fluctuations in their natural environments.

## CONFLICT OF INTEREST

The authors declare no competing interests.

## AUTHOR CONTRIBUTIONS

P.K.P., J.Y., S.A. and N.V. performed the experiments. J.Y. analyzed sequencing data and N.O. and S.A. analyzed the metabolite data. A.R.F., Z.N. and R.A.E.L. supervised the experiments. R.A.E.L. and Z.N. wrote the manuscript and acquired funding.

## References

[pld3186-bib-0001] Busch, F. A. , Sage, R. F. , & Farquhar, G. D. (2018). Plants increase CO_2_ uptake by assimilating nitrogen via the photorespiratory pathway. Nat Plants, 4, 46–54. 10.1038/s41477-017-0065-x 29229957

[pld3186-bib-0002] Callahan, H. S. , & Pigliucci, M. (2002). Shade‐induced plasticity and its ecological significance in wild populations of *Arabidopsis thaliana* . Ecology, 83, 1965–1980. 10.1890/0012-9658(2002)083[1965:SIPAIE]2.0.CO;2

[pld3186-bib-0003] De Pessemier, J. , Chardon, F. , Juraniec, M. , Delaplace, P. , & Hermans, C. (2013). Natural variation of the root morphological response to nitrate supply in *Arabidopsis thaliana* . Mechanisms of Development, 130, 45–53. 22683348 10.1016/j.mod.2012.05.010

[pld3186-bib-0004] Drew, M. C. , Saker, L. R. , & Ashley, T. W. (1973). Nutrient Supply and the Growth of the Seminal Root System in Barley: I. The effect of nitrate concentration on the growth of axes and laterals. Journal of Experimental Botany, 24, 1189–1202. 10.1093/jxb/24.6.1189

[pld3186-bib-0005] Forde, B. G. (2014). Nitrogen signalling pathways shaping root system architecture: An update. Current Opinion in Plant Biology, 21, 30–36. 10.1016/j.pbi.2014.06.004 24997289

[pld3186-bib-0006] Forde, B. G. , & Lea, P. J. (2007). Glutamate in plants: Metabolism, regulation, and signalling. Journal of Experimental Botany, 58, 2339–2358. 10.1093/jxb/erm121 17578865

[pld3186-bib-0007] Giehl, R. F. H. , Gruber, B. D. , & von Wiren, N. (2014). It’s time to make changes: Modulation of root system architecture by nutrient signals. Journal of Experimental Botany, 65, 769–778. 10.1093/jxb/ert421 24353245

[pld3186-bib-0008] Gifford, M. L. , Dean, A. , Gutierrez, R. A. , Coruzzi, G. M. , & Birnbaum, K. D. (2008). Cell‐specific nitrogen responses mediate developmental plasticity. Proceedings of the National Academy of Sciences of the United States of America, 105, 803–808. 10.1073/pnas.0709559105 18180456 PMC2206617

[pld3186-bib-0009] Hachiya, T. , & Sakakibara, H. (2017). Interactions between nitrate and ammonium in their uptake, allocation, assimilation, and signaling in plants. Journal of Experimental Botany, 68, 2501–2512. 28007951 10.1093/jxb/erw449

[pld3186-bib-0010] Jia, Z. T. , Giehl, R. F. H. , Meyer, R. C. , & Altmann, T. , von Wiren, N. (2019) Natural variation of BSK3 tunes brassinosteroid signaling to regulate root foraging under low nitrogen. Nature Communications, 10(1), 2378. 10.1038/s41467-019-10331-9 PMC6542857 31147541

[pld3186-bib-0011] Kiba, T. , & Krapp, A. (2016). Plant nitrogen acquisition under low availability: Regulation of uptake and root architecture. Plant and Cell Physiology, 57, 707–714. 10.1093/pcp/pcw052 27025887 PMC4836452

[pld3186-bib-0012] Lark, R. M. , Milne, A. E. , Addiscott, T. M. , Goulding, K. W. T. , Webster, C. P. , & O'Flaherty, S. (2004). Scale‐ and location‐dependent correlation of nitrous oxide emissions with soil properties: An analysis using wavelets. European Journal of Soil Science, 55, 611–627. 10.1111/j.1365-2389.2004.00620.x

[pld3186-bib-0013] Lisec, J. , Romisch‐Margl, L. , Nikoloski, Z. , Piepho, H. P. , Giavalisco, P. , Selbig, J. , … Willmitzer, L. (2011). Corn hybrids display lower metabolite variability and complex metabolite inheritance patterns. The Plant Journal, 68, 326–336. 10.1111/j.1365-313X.2011.04689.x 21707803

[pld3186-bib-0014] Lu, Y. , & Sharkey, T. D. (2006). The importance of maltose in transitory starch breakdown. Plant, Cell and Environment, 29, 353–366. 10.1111/j.1365-3040.2005.01480.x 17080591

[pld3186-bib-0015] Miller, A. J. , & Cramer, M. D. (2005). Root nitrogen acquisition and assimilation. Plant and Soil, 274, 1–36. 10.1007/s11104-004-0965-1

[pld3186-bib-0016] Nunes‐Nesi, A. , Fernie, A. R. , & Stitt, M. (2010). Metabolic and signaling aspects underpinning the regulation of plant carbon nitrogen interactions. Molecular Plant, 3, 973–996. 10.1093/mp/ssq049 20926550

[pld3186-bib-0017] Pigliucci, M. , & Schlichting, C. D. (1996). Reaction norms of Arabidopsis. 4. Relationships between plasticity and fitness. Heredity, 76, 427–436. 8666543 10.1038/hdy.1996.65

[pld3186-bib-0018] Pigliucci, M. , Schlichting, C. D. , & Whitton, J. (1995). Reaction norms of arabidopsis. 2. response to stress and unordered environmental variation. Functional Ecology, 9, 537–547.

[pld3186-bib-0019] Pigliucci, M. , Whitton, J. , & Schlichting, C. D. (1995). Reaction Norms of Arabidopsis. 1. Plasticity of Characters and Correlations across Water, Nutrient and Light Gradients. Journal of Evolutionary Biology, 8, 421–438.

[pld3186-bib-0020] Plotner, B. , Nurmi, M. , Fischer, A. , Watanabe, M. , Schneeberger, K. , Holm, S. , … Laitinen, R. A. E. (2017). Chlorosis caused by two recessively interacting genes reveals a role of RNA helicase in hybrid breakdown in *Arabidopsis thaliana* . The Plant Journal, 91, 251–262. 28378460 10.1111/tpj.13560

[pld3186-bib-0021] Rachmilevitch, S. , Cousins, A. B. , & Bloom, A. J. (2004). Nitrate assimilation in plant shoots depends on photorespiration. Proceedings of the National Academy of Sciences of the United States of America, 101, 11506–11510. 10.1073/pnas.0404388101 15272076 PMC509230

[pld3186-bib-0022] Roessner, U. , Luedemann, A. , Brust, D. , Fiehn, O. , Linke, T. , Willmitzer, L. , & Fernie, A. R. (2001). Metabolic profiling allows comprehensive phenotyping of genetically or environmentally modified plant systems. The Plant Cell, 13, 11–29. 10.1105/tpc.13.1.11 11158526 PMC2652711

[pld3186-bib-0023] Schauer, N. , Steinhauser, D. , Strelkov, S. , Schomburg, D. , Allison, G. , Moritz, T. , … Kopka, J. (2005). GC‐MS libraries for the rapid identification of metabolites in complex biological samples. FEBS Letters, 579, 1332–1337. 10.1016/j.febslet.2005.01.029 15733837

[pld3186-bib-0024] Stitt, M. , & Fernie, A. R. (2003). From measurements of metabolites to metabolomics: An 'on the fly' perspective illustrated by recent studies of carbon‐nitrogen interactions. Current Opinion in Biotechnology, 14, 136–144. 10.1016/S0958-1669(03)00023-5 12732314

[pld3186-bib-0025] Stitt, M. , & Krapp, A. (1999). The interaction between elevated carbon dioxide and nitrogen nutrition: The physiological and molecular background. Plant Cell and Environment, 22, 583–621. 10.1046/j.1365-3040.1999.00386.x

[pld3186-bib-0026] Swiadek, M. , Proost, S. , Sieh, D. , Yu, J. , Todesco, M. , Jorzig, C. , … Laitinen, R. A. (2017). Novel allelic variants in *ACD6* cause hybrid necrosis in local collection of *Arabidopsis thaliana* . New Phytologist, 213, 900–915. 27588563 10.1111/nph.14155

[pld3186-bib-0027] Thalmann, M. , & Santelia, D. (2017). Starch as a determinant of plant fitness under abiotic stress. New Phytologist, 214, 943–951. 10.1111/nph.14491 28277621

[pld3186-bib-0028] Verkroost, A. W. M. , & Wassen, M. J. (2005). A simple model for nitrogen‐limited plant growth and nitrogen allocation. Annals of Botany, 96, 871–876. 10.1093/aob/mci239 16100225 PMC4247053

