# Supplementary material for: Plasticity in metabolism underpins local responses to nitrogen in Arabidopsis thaliana populations
Source: Plant Direct. 2019 Nov 29;3(11):e00186. doi: 10.1002/pld3.186 (PMC6884650; doi:10.1002/pld3.186)
Supplement: Supplementary file 1 [file PLD3-3-e00186-s001.pdf]

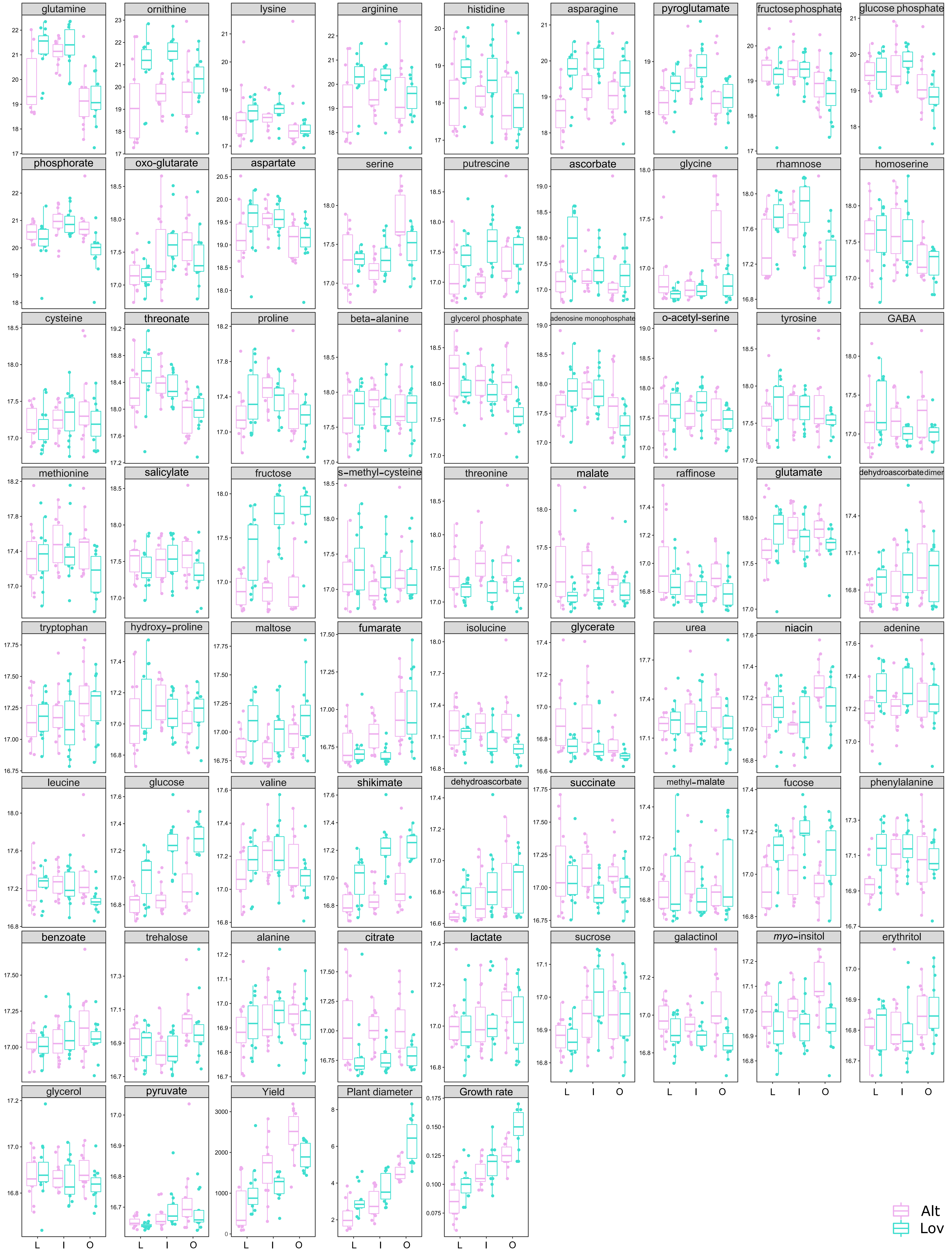


**Figure S1** Box plots for metabolic, growth, and yield traits in the Alt and Lov- populations. Each panel shows box plots of trait means over the replicates for the 15 individuals in the two populations under the three N conditions. On the Y-axis, the metabolite levels are expressed in arbitrary units (a.u.) relative to a standard, plant diameter in cm, growth rate in cm per day, and yield is quantified by the number of seeds per plant (see Supplemental information). The x-axis represents the limited (L), intermediate (I) and optimal (O) N conditions.
