# Supplementary material for: Plasticity in metabolism underpins local responses to nitrogen in Arabidopsis thaliana populations
Source: Plant Direct. 2019 Nov 29;3(11):e00186. doi: 10.1002/pld3.186 (PMC6884650; doi:10.1002/pld3.186)
Supplement: Supplementary file 2 [file PLD3-3-e00186-s002.pdf]

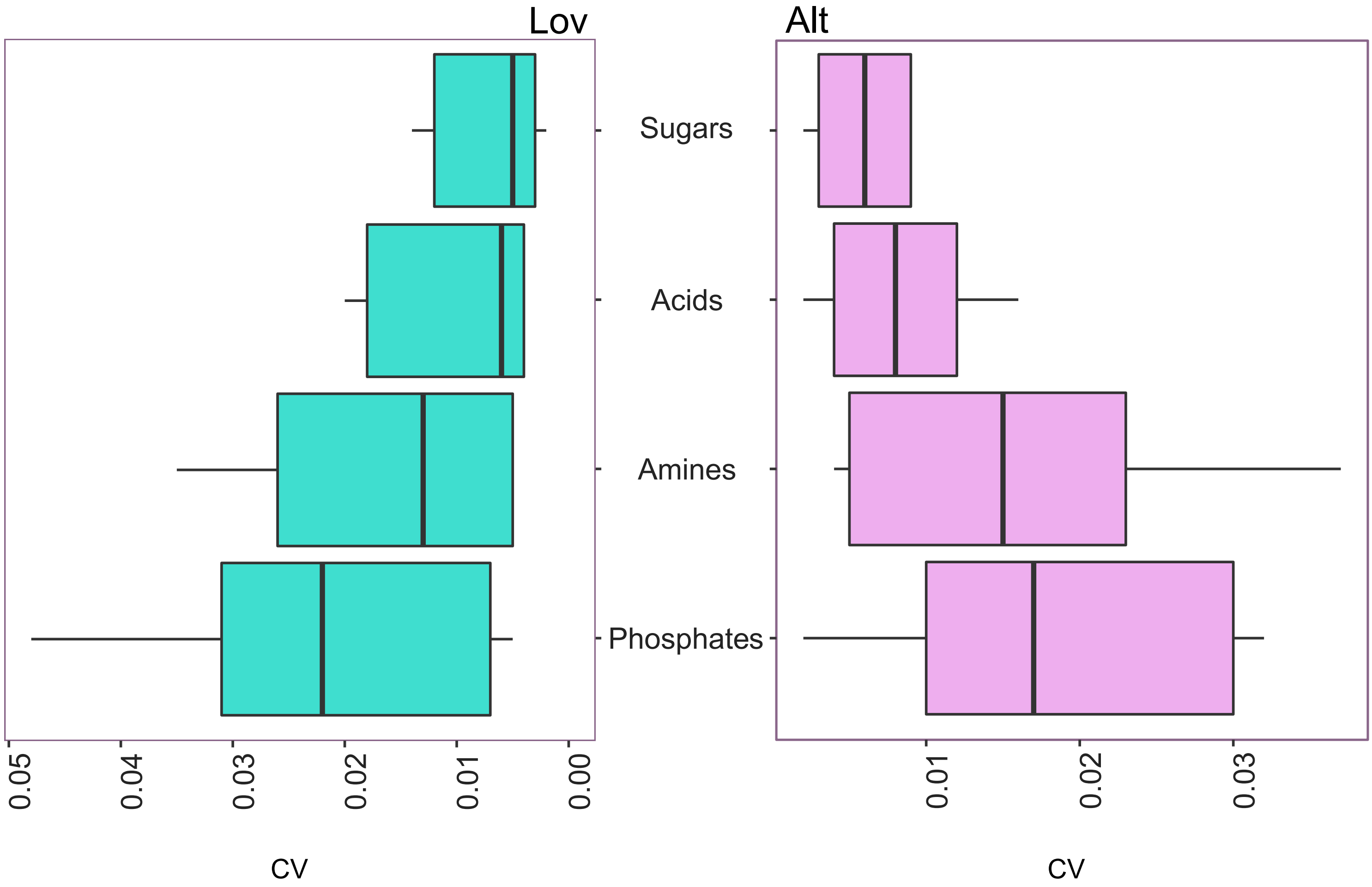

Figure S2 Comparison of plasticity among metabolite classes in the Alt and Lov-populations. The plasticity in the metabolite classes is given by the distribution of CVs of the comprising metabolites, depicted as box plots. From the 65 primary metabolites, 25 were amines, 19 acids, 12 sugars and 4 phosphates.
