# Supplementary material for: Plasticity in metabolism underpins local responses to nitrogen in Arabidopsis thaliana populations
Source: Plant Direct. 2019 Nov 29;3(11):e00186. doi: 10.1002/pld3.186 (PMC6884650; doi:10.1002/pld3.186)
Supplement: Supplementary file 4 [file PLD3-3-e00186-s004.pdf]

Table S2. P-values showing significance of the metabolite level distributions between the two *A. thaliana* populations Alt and Lov. (Kolmogorov-Smirnow (KS) test, p-value adjusted by the Benjamini-Hochberg procedure)

**Metabolite**	**p-value**
adenine	0.0109472163152025
adenosine monophosphate	0.583275973218677
alanine	0.83141213032566
beta -alanine	0.465055752764957
arginine	3,80E+09
ascorbate	0.00132414542845349
asparagine	3,26E+13
aspartate	0.528370002123929
benzoate	0.583275973218677
citrate	1,10E+07
s-methyl-cysteine	0.139440200441938
cysteine	0.725781920165751
dehydroascorbate	0.0151667415074377
dehydroascorbate dimer	0.215403905817136
erythritol	0.83141213032566
fructose	0
fructose phosphate	0.139440200441938
fucose	1,79E+03
fumarate	0.0282558981268109
GABA	0.583275973218677
galactinol	1,55E+08
glucose	0
glucose phosphate	0.638094475997111
glutamate	0.638094475997111
glutamine	0.0521433239277556
oxo -glutarate	0.638094475997111
glycerate	1,55E+08
glycerol	0.319770786854152
glycerol phosphate	0.000236603041627607
glycine	1,88E+07
histidine	0.00281296744905836
homoserine	0.387854796510809
myo- insitol	2,39E+09
isolucine	0.000236603041627607
lactate	0.583275973218677
leucine	0.528370002123929
lysine	0.00575954424620824
methyl-malate	0.0686332341046385
malate	3,21E+07
maltose	5,45E+07
methionine	0.0894214741967467
niacin	0.784024149856424
ornithine	2,13E+05
phenylalanine	0.00812520505066833
phosphorate	0.000571591083064121
proline	0.528370002123929
hydroxy-proline	0.171979130273253
putrescine	1,30E+03
pyroglutamate	0.0109472163152025
pyruvate	0.83141213032566
raffinose	0.171979130273253
rhamnose	0.00281296744905836
salicylate	0.139440200441938
serine	0.00575954424620824
o-acetyl serine	0.0282558981268109
shikimate	0
succinate	0.000236603041627607
sucrose	0.319770786854152
trehalose	0.266927561225869
threonate	0.638094475997111
threonine	1,01E+03
tryptophan	0.978035935315962
tyrosine	0.139440200441938
urea	0.784024149856424
valine	0.784024149856424
