# Supplementary material for: Plasticity in metabolism underpins local responses to nitrogen in Arabidopsis thaliana populations
Source: Plant Direct. 2019 Nov 29;3(11):e00186. doi: 10.1002/pld3.186 (PMC6884650; doi:10.1002/pld3.186)
Supplement: Supplementary file 3 [file PLD3-3-e00186-s003.pdf]

Table S1 The percentage (%) of genetic similarity between the individuals of Alt and Lov populations of *A. thaliana* based on 2171 informative markers from RADseq data from Swiadek et al. 2017 and Plötner et al. 2017.

	Alt13	Alt16	Alt17	Alt21	Alt22	Alt25	Alt29	Alt30	Alt34	Alt19	Alt22	Alt27	Alt35	Alt40
**Alt10**	71.5	78.7	70.8	79.6	79.0	85.3	78.0	65.0	77.5	87.9	80.5	70.9	78.2	86.6
**Alt13**		75.5	75.3	79.1	71.8	72.3	70.8	67.6	71.8	74.2	76.0	75.3	71.0	73.0
**Alt16**			76.2	80.5	76.5	76.8	75.8	69.2	76.4	80.2	81.1	76.2	76.0	77.1
**Alt17**				71.0	77.4	71.8	78	83.2	76.9	78.1	83.2	99.7	77.6	72.5
**Alt21**					73.3	76.6	69.8	65.9	73	77.2	76.9	71.1	69.7	77.7
**Alt22**						74.4	75.7	73.9	86.2	76.6	75.6	77.3	75.5	75
**Alt25**							73.6	65.6	75.4	79.9	76.7	71.9	73.9	97.5
**Alt29**								70.2	77.3	84.3	80.9	77.6	98.6	74.4
**Alt30**									69.9	70.7	72.8	83.4	70.1	65.6
**Alt34**										76.2	76.3	76.8	77.8	76.5
**Alt19**											91.0	77.8	84	80.8
**Alt22**												82.9	80.5	77.6
**Alt27**													77.4	72.5
**Alt35**														74.7

	Lov07	Lov13	Lov19	Lov25	Lov31	Lov37	Lov43	Lov49	Lov55	Lov61	Lov67	Lov74	Lov79	Lov85
**Lov01**	98.9	99.2	99.9	99.6	99.8	98.9	98.9	99.7	99.6	99.8	99.2	99.8	99.8	99.4
**Lov07**		98.6	98.9	98.7	98.7	99.2	99.2	98.7	98.7	98.9	98.6	98.8	98.8	98.8
**Lov13**			99.2	99.4	99.1	99.0	99.0	99.1	99.3	99.1	99.7	99.1	99.1	99.7
**Lov19**				99.6	99.8	98.9	98.9	99.7	99.7	99.8	99.2	99.8	99.8	99.4
**Lov25**					99.5	99.1	99.0	99.6	99.7	99.6	99.3	99.7	99.5	99.4
**Lov31**						98.7	98.7	99.7	99.5	99.8	99.1	99.7	99.7	99.3
**Lov37**							99.7	98.9	99.0	98.9	98.9	99	98.8	99.1
**Lov43**								98.8	99.0	98.9	98.9	98.9	98.8	99.1
**Lov49**									99.4	99.8	99	99.8	99.8	99.2
**Lov55**										99.7	99.4	99.5	99.5	99.5
**Lov61**											99.1	99.9	99.9	99.4
**Lov67**												99.1	99.1	99.6
**Lov74**													99.8	99.3
**Lov79**														99.3
