# Supplementary material for: Plasticity in metabolism underpins local responses to nitrogen in Arabidopsis thaliana populations
Source: Plant Direct. 2019 Nov 29;3(11):e00186. doi: 10.1002/pld3.186 (PMC6884650; doi:10.1002/pld3.186)
Supplement: Supplementary file 5 [file PLD3-3-e00186-s005.pdf]

Dear Dr. Baxter and Dr. Moghe,

We would like to thank you and the referee for the additional suggestions to improve our manuscript titled "Plasticity in metabolism underpins local responses to nitrogen in *Arabidopsis thaliana* populations". We have now addressed the concerns and revised the manuscript accordingly. All the changes are also visible in the manuscript file with track changes.

The more detailed response to yours and the referee comments (in blue) is found below.

Sincerely yours,

Roosa Laitinen and Zoran Nikoloski

In Reviewer 2's review, please address the points marked by ***

Addressing remaining comments is optional, and will not affect subsequent editorial decision.

Also, please fix the title of Table S1 (% genetic similarity)
#########

To upload your revision, please click the link below.
https://plantdirect.msubmit.net/cgi-bin/main.plex?el=A2Lr2IC4B6iHM7I6A9ftdRogdjGgy0pdWHHu70iZKXgZ

In order to provide as timely a service as possible, we ask that your revision is resubmitted within three months after receipt of this request. If an extension is needed, please send a request, along with a brief explanation, to the editorial office at plantdirect@wiley.com .

Please note that, in addition to publishing reviewer comments, the author's responses to review comments will also be published alongside the final version of the paper. If you would not like the author's responses to be published, please contact the editorial office at plantdirect@wiley.com .

Thank you very much for giving us an opportunity to review your work. I look forward to receiving the next version.

Sincerely,

Gaurav Moghe
Ivan Baxter
Editor, Plant Direct

--- Reviewer comments:
Reviewer #2:

1. The setup of this study, measuring plasticity in two Arabidopsis populations that differ in intrapopulation genetic diversity, is well-poised to investigate the importance of genetic variation and phenotypic plasticity versus phenotypic stability/flexibility in local adaptation. You briefly present these ideas in the introduction, but how/whether the results support one strategy over another was not always clear and it was difficult for me to maintain the narrative thread. Here are a couple of references that may be useful in this regard (though there are certainly others): i) Scheiner, S. M., & Goodnight, C. J. (1984). The comparison of phenotypic plasticity and genetic variation in

populations of the grass Danthonia spicata. Evolution, 38(4), 845-855 and ii) Scheiner, S. M. (1993). Genetics and evolution of phenotypic plasticity. Annual review of ecology and systematics, 24(1), 35-68.

We agree with the reviewer that linking the phenotypic plasticity to genetic variation and also to local adaptation is an highly interesting question. However, our experimental setup was not able to address these points and therefore we have not emphasized them in our narrative.

2. How does the response to different N conditions relate to environmental variation between Southern Germany and Northern Sweden? You mention that the Lov site is relatively undisturbed while the Alt site is highly disturbed. Can you speak to how that is expected to correlate with N availability?

We agree with the reviewer that it would be interesting to relate our findings to the actual N availability in the populations. However, the different growth environments of these sites could imply that they could also differ in their N availability but without soil analysis these are only speculations. We refrained from inclusion of a discussion of this point.

3. How does the genetic diversity within Alt and Lov compare to genetic variation between these populations? Other Arabidopsis populations? Across the species? It seems like there is a ton of genetic diversity in the Alt population. Is this true? Are the SNPs found within Alt and Lov unique or shared? Is it known whether populations of Arabidopsis or other weedy species occupying disturbed sites tend to harbor greater genetic diversity?

We thank the reviewer for this point. In our analysis we did not investigate how the genetic variation within compares to the genetic variation between the two populations. In comparison to the other accessions, the genetic diversity in Alt-population is typical for the area from where it is collected from (Bomblies et al. 2010). Since our present design does not allow for directly linking variability in plasticity with genetic variability, we did not include the mentioned interesting questions in our manuscript as that draws attention from the main points we wanted to emphasize.

4. How much residual heterozygosity was present in Lov versus Alt accessions after a single generation of selfing in the greenhouse? I looked at your citations and found that heterozygosity in the Lov population was measured at 1-2%. Considering the Alt population is much more genetically diverse, it seems reasonable that higher residual heterozygosity would be apparent as well. Given that the accessions only underwent a single round of selfing in the greenhouse before being used in this study, then the Alt accessions would represent segregating populations, rather than inbred lines. The confounding genetic diversity within the Alt accessions would likely inflate the apparent plasticity. Can you briefly address this possibility, either in the methods or results and discussion?
** This is an important point that needs to be clarified in the manuscript text

We thank the reviewer for this important point. We have now added information of the heterozygosity in the population and made it clear in both result and discussion and in the methods that the accessions used in this study are the next generation of the genotyped accessions. Therefore, the heterozygous loci are segregating in the population. Yet, we also stress that our aim was not to link the plasticity to certain genetic differences but rather to highlight that even the genetically diverse populations showed similar plasticity in specific metabolites but different amount

in some other. Further, an interesting finding was that even the genetically very similar individuals in Lov-populations showed high plasticity in specific metabolites.

The following text was added to the results and discussion:

“Four out of the five polymorphic SNPs in Lov- population were heterozygous leaving only a single polymorphic nucleotide in only one of the individual. The overall heterozygosity in Alt-population was low (He=0.199) but higher than in Lov- population (0.009). It is notified that seeds used in this study are the next generation from the selfed accessions that were genotyped and in these the heterozygous loci are segregating yet influencing the genetic diversity. Nevertheless, the Alt-population was genetically more diverse than in the Lov- population.”

The following clarification was added to the methods:

“Degree of heterozygosity (He index) was calculated using package ‘pegas’ in statistical environment R.”

5. Lines 83-90: The way plasticity is used and defined seems to be inconsistent here. Phenotypic plasticity refers to the ability of a single genotype to produce different phenotypes under different environmental conditions, which is how you use the term in lines 83-87. Then, in the next sentence you say that the 'genetically more diverse Alt population was also more plastic than the Lov population', referring to Figure 1. There is very little genetic diversity in the Lov population, such that those accessions are effectively clones and can be treated as a single genotype. However, the Alt accessions surely represent distinct genotypes. I think that what you mean in lines 87-90 is that accessions from the Alt population display a greater range of plastic responses (i.e. some Alt accessions are highly plastic and others are not) compared to the Lov population. If that is what you mean, then it would be more accurate to say that the Alt population harbors genetic polymorphisms that influence phenotypic plasticity. On the other hand, if what you mean is that the Alt accessions tend to display higher phenotypic plasticity across the board, then that makes me wonder about residual heterozygosity (see comment #3). Honestly, I cannot tell by looking at Figures 1, S1 and S2 whether CV is more variable in the Alt population or just simply higher than the Lov population. Parsing this out would make the results more interpretable for the reader and likely more impactful. ** Please only address this comment in the results or discussion. No need to make new figures

The reviewer is correct that the plasticity is a trait of an individual not population. It is also true that the Alt individuals show larger range of plasticity than Lov-populations. To quantify the amount of plasticity among individuals in the populations we have used the average plasticity of all individuals. We have clarified the text when we talk about the average plasticity among the individuals and when we talk the range of plasticity.

6. I wonder how Figure S1 would look if the box plots were instead drawn as reaction norm plots with 30 lines total, representing the Alt and Lov accessions. It may make the figure too busy, but it would be nice to see how each accession responds to the different N treatments. With the box plots, it is impossible to tell how the accessions vary in their plastic response.

7. Can you include a methods sections to describe the population genetic analyses conducted for this study? In lines 85-87, you mention that there were 2171 informative SNPs for Lov, but do not explain

where that number comes from. Indeed, it differs from the 2591 informative SNPs described in Plotner et al., 2017 that you cite as a reference for this information, suggesting that further analyses were conducted for this study. In this methods section, please describe the criteria used to designate SNPs as informative or uninformative. Also, describe how genetic diversity was calculated.
** Please address this

We thank the reviewer for noticing the absence of genetic analysis in the methods section. We have now added the following description to the methods

"For the genetic analysis, the RAD-seq data from Plötner et al., 2017 and Swiadek et al., 2016 was used to investigate the genetic differences among populations First RADseq data was filtered using 10 x coverage in all samples and to contain at least one polymorphic SNP. The resulting in 2171 SNPs among the populations were assigned as informative and were used for further analysis. For percentage (%) of sequence similarity these SNPs were compared to each other using MEGALIGN."

8. Lines 100-101: This statement is not clear. I think what you means here is that in cases where the CV is similar between Alt and Lov, the shape of the phenotypic response curve across the three N treatments is similar. However, the wording is vague and makes it difficult for the reader to grasp the meaning.

We have modified this sentence accordingly.

9. Lines 183-185: Please expand this section to describe how the accessions used in this study were generated. In the response document, you mention that seeds were collected from field-grown plants, then grown in the greenhouse for one generation to produce seeds used in this study. However, the materials and methods section needs to be updated to reflect this. Please include the year(s) that the round of selfing in the greenhouse occurred and then when those seeds were used for this study.

We have added information of the generation of the accessions.

Reviewer #3:

I do not have other comments.
